# Prevalence, Maternal Risk Factors, and Neonatal Outcomes of Early-Onset Neonatal Septicemia: A Retrospective Study

**DOI:** 10.7759/cureus.110821

**Published:** 2026-06-14

**Authors:** Hansraj Brijlal Khurana, Ritika Khurana

**Affiliations:** 1 Department of Pediatrics, Government Medical College, Hingoli, Hingoli, IND; 2 Department of Obstetrics and Gynecology, Surya Hospital, Pune, IND

**Keywords:** early onset neonatal septicemia, low birth weight, neonatal intensive care unit, neonatal sepsis, preterm birth

## Abstract

Introduction

Neonatal infections continue to pose major challenges in intensive care settings. This study aimed to evaluate the prevalence, maternal risk factors, neonatal characteristics, and clinical outcomes associated with early-onset neonatal septicemia in neonates admitted to the neonatal intensive care unit.

Materials and methods

This retrospective observational cohort study was conducted in the neonatal intensive care unit at Government Medical College, Hingoli, India, using medical records of neonates admitted between January 2019 and December 2024. A total of 128 neonates aged 0-28 days were included in the study. Maternal variables, such as preterm delivery, prolonged membrane rupture, intrapartum fever, chorioamnionitis, and maternal urinary tract infection, were assessed. Neonatal variables, including birth weight, Apgar score, resuscitation at birth, and clinical outcomes, were evaluated. Early-onset neonatal septicemia was defined as clinical signs of sepsis occurring within the first 72 hours of life, with a positive blood culture and/or positive sepsis screening parameters. Statistical analysis was performed, and statistical significance was set at p < 0.05.

Results

The prevalence of early-onset neonatal septicemia was found in 21 neonates (16.4%), including proven sepsis in 13 neonates (10.2%) and probable sepsis in eight neonates (6.2%). Preterm delivery, prolonged membrane rupture, intrapartum fever, and chorioamnionitis were significantly associated with early-onset neonatal septicemia (p < 0.05). Low birth weight was observed in 17 neonates (81.0%) with sepsis compared with 41 neonates (38.3%) without sepsis (p < 0.001). Mechanical ventilation was required in 11 neonates (52.4%), while in-hospital mortality was observed in four neonates (19.0%) with early-onset neonatal septicemia. Multivariate logistic regression analysis identified prolonged rupture of membranes (aOR: 4.67), intrapartum fever (aOR: 3.89), preterm delivery (aOR: 3.21), low birth weight (aOR: 2.98), and a five-minute Apgar score <7 (aOR: 2.42) as independent predictors of early-onset neonatal septicemia.

Conclusion

Early-onset neonatal septicemia remains a significant cause of neonatal morbidity and mortality in neonatal intensive care units. Prolonged rupture of membranes, intrapartum fever, preterm delivery, low birth weight, and a low five-minute Apgar score were identified as independent predictors of early-onset neonatal septicemia. Early identification of high-risk neonates and timely management strategies may improve neonatal outcomes.

## Introduction

Early-onset neonatal septicemia is one of the leading causes of neonatal morbidity and mortality worldwide, particularly in developing countries where the burden of neonatal infections remains high [1]. Early-onset neonatal septicemia usually manifests within the first 72 hours of life and is commonly acquired through vertical transmission of microorganisms from the mother during labor or delivery. Prematurity, prolonged membrane rupture, maternal fever, chorioamnionitis, and low birth weight are recognized contributors to neonatal susceptibility to sepsis [2]. Despite advancements in neonatal intensive care and antimicrobial therapy, early diagnosis and timely intervention remain challenging because clinical manifestations are often nonspecific and overlap with those of other neonatal conditions.

The incidence and outcomes of early-onset neonatal septicemia vary considerably across geographical regions and healthcare settings. Tertiary care hospitals, especially neonatal intensive care units, manage critically ill neonates and therefore encounter a substantial burden of neonatal sepsis [3]. Culture-positive sepsis remains the gold standard for diagnosis; however, many neonates with clinical features suggestive of sepsis may have negative blood cultures because of prior maternal antibiotic exposure, inadequate blood sample volume, or low bacterial load [4]. Consequently, sepsis screening parameters, such as total leukocyte count, absolute neutrophil count, immature-to-total neutrophil ratio, and C-reactive protein level, are frequently used to support diagnosis in clinically suspected cases [5].

Maternal and neonatal risk factors play a critical role in determining the occurrence and progression of early-onset neonatal septicemia [6]. The identification of modifiable maternal risk factors and neonatal predictors may facilitate early screening, prompt treatment, and improve neonatal outcomes [7]. Furthermore, evaluating neonatal complications, such as prolonged hospital stay, mechanical ventilation requirement, vasopressor support, and mortality, can provide valuable insights into the burden of disease and resource utilization in intensive care settings.

Therefore, the present study was conducted to evaluate the prevalence, maternal risk factors, and outcomes associated with early-onset neonatal septicemia in neonates admitted to the neonatal intensive care unit of a tertiary care hospital. The objectives of this study were to determine the prevalence of early-onset neonatal septicemia, assess maternal and neonatal risk factors associated with the condition, identify independent predictors of early-onset neonatal septicemia, and evaluate neonatal outcomes, including the duration of hospital stay, need for respiratory and vasopressor support, complications, and in-hospital mortality.

## Materials and methods

Study design and setting

This retrospective observational cohort study was conducted at Government Medical College, Hingoli, India. This study involved a retrospective review and analysis of the hospital medical records of neonates admitted between 2019 and 2024. The methodology was prepared in accordance with the Strengthening the Reporting of Observational Studies in Epidemiology guidelines for observational studies [8]. The medical records of 396 neonates admitted to the neonatal intensive care unit between January 1, 2019, and December 31, 2024, were retrospectively reviewed for eligibility assessment and data extraction. The study was approved by the Ethics Committee (approval no. 1001/2025; dated May 20, 2025).

Study population

**Figure 1:**
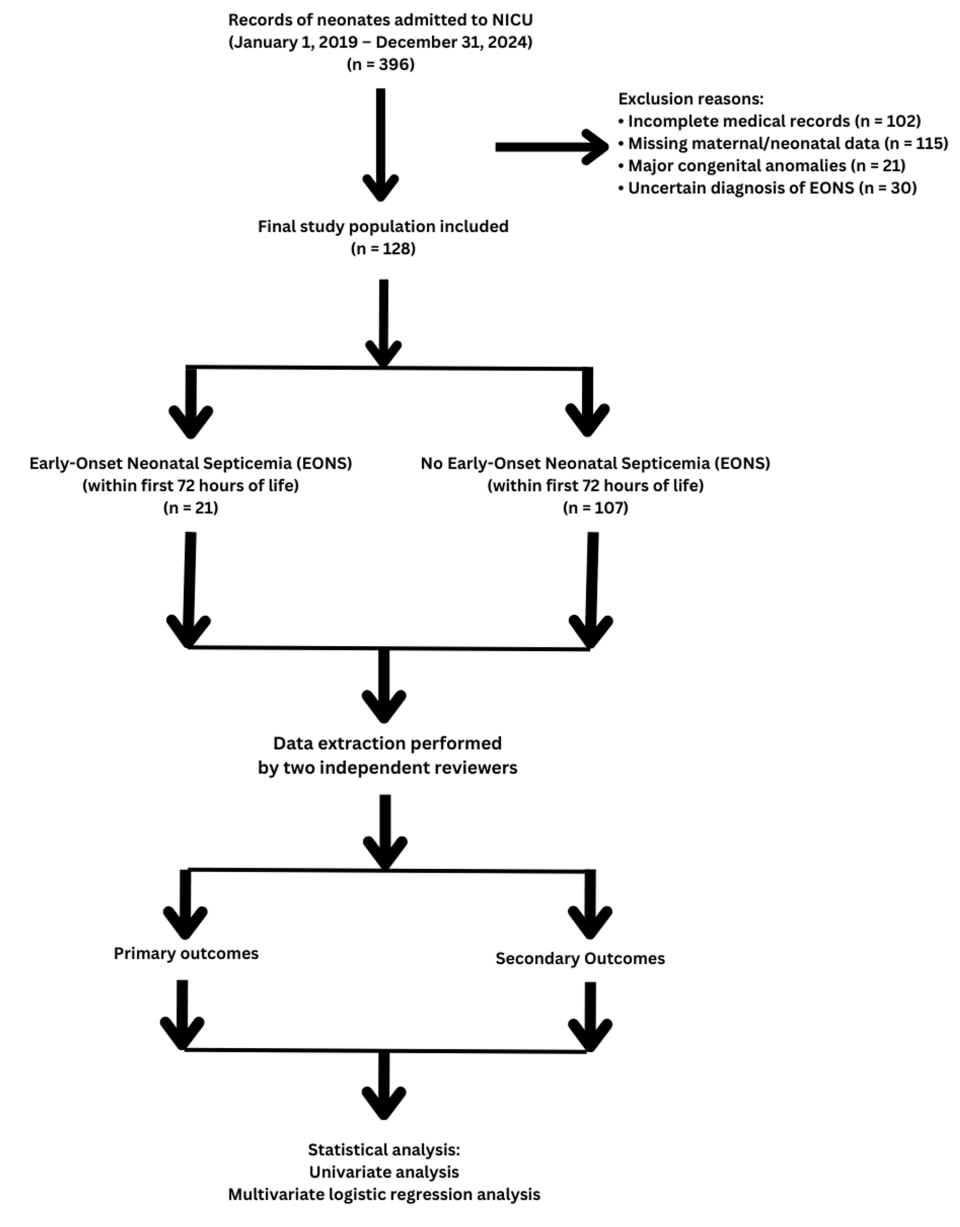
Study flowchart.

The study population consisted of all neonates aged 0-28 days admitted to the neonatal intensive care unit during the study period. Neonates delivered at the same institution and those referred from other healthcare facilities were included in the study population (Figure 1).

Sample size estimation

A priori sample size estimation was performed using G*Power statistical software (Heinrich Heine University Düsseldorf, Düsseldorf, Germany). The calculation was based on a previous study, and the expected prevalence in the study population was assumed to be 20%, corresponding to an anticipated difference of 4% [9]. Using the z-test family for the comparison of two proportions with a two-tailed analysis, a significance level of 5% and statistical power of 80% were applied. The minimum estimated sample size was 128 neonates.

Eligibility criteria

Neonates diagnosed with early-onset neonatal septicemia, defined as clinical signs and symptoms of sepsis occurring within the first 72 hours of life and supported either by a positive blood culture or by positive sepsis screening parameters in the presence of suggestive clinical features, were included. Only neonates with complete maternal and neonatal medical records available for review and data extraction were considered eligible for inclusion in the study. Neonates with incomplete or missing medical records, which prevented adequate data extraction, were excluded from this study. Neonates with major congenital anomalies and those diagnosed exclusively with late-onset sepsis occurring after 72 hours of life without concurrent early-onset neonatal septicemia were also excluded. Cases in which the diagnosis of early-onset neonatal septicemia could not be clearly established from available records were excluded from the final analysis.

Data collection procedure

A structured case report form was developed before the initiation of the study to ensure uniformity and consistency in data collection (Appendix 1). The form was piloted using 10-15 medical records to refine operational definitions and improve inter-reviewer reliability. Data were collected independently by two trained reviewers (HBK and RK) from electronic medical records and paper-based admission registers. 

Maternal variables assessed

The maternal variables assessed in this study included maternal age, parity, gestational age at delivery, and mode of delivery. Maternal risk factors such as prolonged rupture of membranes, defined as rupture of membranes lasting more than 18 hours before delivery; maternal intrapartum fever greater than or equal to 38°C; clinical or histopathological chorioamnionitis; maternal urinary tract infection during pregnancy; and maternal Group B *Streptococcus* colonization status were recorded wherever available. Routine antenatal screening for maternal Group B *Streptococcus* colonization was not uniformly performed during the study period; therefore, this variable was available only for a subset of cases, and missing data were excluded from comparative analysis for that specific parameter. Information regarding the administration of intrapartum antibiotic prophylaxis and associated maternal medical conditions, such as diabetes mellitus and hypertensive disorders of pregnancy, was also obtained from medical records.

Neonatal variables assessed

Neonatal variables evaluated in the study included birth weight, sex, Apgar scores at one minute and five minutes, requirement of resuscitation at birth, presence of perinatal asphyxia, and meconium-stained amniotic fluid [10]. Birth weight was categorized as low, very low, or extremely low according to standard definitions. Perinatal asphyxia was defined as a five-minute Apgar score < 7 and/or umbilical cord pH < 7.1 [11].

Diagnostic criteria and outcome measures

Early-onset neonatal septicemia was defined as the presence of clinical signs and symptoms of sepsis occurring within the first 72 hours of life, supported either by a positive blood culture or by positive sepsis screening parameters in the presence of suggestive clinical features [12]. Sepsis screening parameters included total leukocyte count less than 5000/mm³ or greater than 20,000/mm³, absolute neutrophil count less than 1800/mm³, immature-to-total neutrophil ratio greater than 0.2, micro-ESR greater than 15 mm in the first hour, and C-reactive protein greater than 10 milligrams per liter. Clinical signs and symptoms assessed included respiratory distress, poor feeding, lethargy, jaundice, and temperature instability. Blood culture findings, organism identification, duration of neonatal intensive care unit stay, need for mechanical ventilation, requirement of vasopressor support, and development of complications such as shock, disseminated intravascular coagulation, necrotizing enterocolitis, and in-hospital mortality were recorded as outcome measures.

Statistical analysis

Data obtained from medical records were statistically analyzed using IBM SPSS Statistics for Windows, Version 25 (Released 2017; IBM Corp., Armonk, NY, USA). Descriptive statistics were used to summarize demographic and clinical characteristics. The normality of continuous variables was assessed using the Shapiro-Wilk test. Continuous variables were expressed as means and standard deviations or medians and interquartile ranges, depending on the data distribution, whereas categorical variables were presented as frequencies and percentages. The prevalence of early-onset neonatal septicemia was calculated using 95% confidence intervals. Group comparisons were performed using the chi-square test or Fisher’s exact test for categorical variables and the independent t-test or Mann-Whitney U test for continuous variables. Variables with p < 0.05 in the bivariate analysis were entered into multivariate logistic regression analysis to identify independent predictors of early-onset neonatal septicemia and to calculate adjusted odds ratios with 95% confidence intervals. Statistical significance was set at p < 0.05.

## Results

Table 1 shows the prevalence of early-onset neonatal septicemia among neonates admitted to the neonatal intensive care unit. Among the 128 neonates included in the study, early-onset neonatal septicemia was identified in 21 neonates (16.4%). Proven sepsis with positive blood culture was observed in 13 neonates (10.2%), while probable sepsis based on positive sepsis screening and clinical findings was observed in eight neonates (6.2%). The remaining 107 neonates (83.6%) showed no evidence of early-onset septicemia.

**Table 1:** Prevalence of early onset neonatal septicemia among neonates admitted to the neonatal intensive care unit. Data presented as frequency and percentage, confidence interval presented as 95% confidence interval, n = number of neonates.

Category	Number of neonates, n (%)	95% confidence interval
Early onset neonatal septicemia (proven + probable)	21 (16.4)	(10.5 - 23.7)
Proven sepsis (positive blood culture)	13 (10.2)	(5.6 - 16.8)
Probable sepsis (positive sepsis screen + clinical signs)	8 (6.2)	(2.8 - 11.8)
No early onset neonatal septicemia	107 (83.6)	(76.3 - 89.5)

Table 2 demonstrates the comparison of maternal risk factors between the early-onset neonatal septicemia and non-early-onset neonatal septicemia groups. Preterm delivery was significantly more common among neonates with early-onset neonatal septicemia than among those without early-onset neonatal septicemia (p = 0.003). Prolonged membrane rupture was observed in 14 mothers (66.7%) in the early-onset neonatal septicemia group and in 25 mothers (23.4%) in the non-early-onset neonatal septicemia group (p < 0.001). Intrapartum fever was present in nine mothers (42.9%) in the early-onset neonatal septicemia group compared to 12 mothers (11.2%) in the non-early-onset neonatal septicemia group (p < 0.001). Clinical chorioamnionitis was also significantly associated with early-onset neonatal septicemia and was observed in eight mothers (38.1%) compared to 11 mothers (10.3%) in the non-early-onset neonatal septicemia group (p = 0.001).

**Table 2:** Comparison of maternal risk factors between neonates with and without early-onset neonatal septicemia. Data were presented as mean ± standard deviation (SD) or frequency and percentage. The chi-square test and Fisher’s exact test were used for categorical variables, and the independent t-test was used for continuous variables. *p < 0.05 was considered statistically significant; n = number of neonates; χ² = chi-square test.

Maternal risk factor	Early onset neonatal septicemia (n = 21), n (%)	Non-early onset neonatal septicemia (n = 107), n (%)	Test statistic	p‑value
Maternal age (years), mean ± SD	28.4 ± 5.2	27.9 ± 5.5	t = 0.39	0.697
Primiparity, n (%)	13 (61.9)	58 (54.2)	χ² = 0.43	0.512
Preterm delivery (<37 weeks), n (%)	15 (71.4)	39 (36.4)	χ² = 8.79	0.003*
Cesarean section, n (%)	12 (57.1)	62 (57.9)	χ² = 0.01	0.944
Prolonged rupture of membranes (>18 hours), n (%)	14 (66.7)	25 (23.4)	χ² = 15.36	<0.001*
Intrapartum fever (≥38°C), n (%)	9 (42.9)	12 (11.2)	χ² = 13.69	<0.001*
Clinical chorioamnionitis, n (%)	8 (38.1)	11 (10.3)	χ² = 11.23	0.001*
Maternal urinary tract infection during pregnancy, n (%)	5 (23.8)	18 (16.8)	χ² = 0.60	0.440
Group B *Streptococcus* colonization (known), n (%)	3/8 (37.5)	9/32 (28.1)	Fisher’s	0.677
Intrapartum antibiotic prophylaxis given, n (%)	4 (19.0)	28 (26.2)	χ² = 0.49	0.483

Table 3 presents neonatal characteristics associated with early-onset neonatal septicemia. Low birth weight was significantly more frequent among neonates with early-onset neonatal septicemia than among those without early-onset neonatal septicemia (p < 0.001). A five-minute Apgar score less than seven was observed in nine neonates (42.9%) in the early-onset neonatal septicemia group and 18 neonates (16.8%) in the non-early-onset neonatal septicemia group (p = 0.007). Resuscitation at birth was required in 11 neonates (52.4%) with early-onset neonatal septicemia compared to 26 neonates (24.3%) without early-onset neonatal septicemia (p = 0.008). No statistically significant association was observed between sex and meconium-stained amniotic fluid.

**Table 3:** Association of neonatal characteristics with early-onset neonatal septicemia. Data were presented as mean ± standard deviation (SD) or frequency and percentage. The chi-square test was used for categorical variables, and the independent t-test was used for continuous variables. *p < 0.05 was considered statistically significant; n = number of neonates; χ² = chi-square test.

Neonatal characteristic	Early-onset neonatal septicemia (n = 21), n (%)	Non-early-onset neonatal septicemia (n = 107), n (%)	Test statistic	p‑value
Birth weight (grams), mean ± SD	1920.0 ± 580.0	2680.0 ± 710.0	t = 4.62	< 0.001*
Low birth weight (<2500 g), n (%)	17 (81.0)	41 (38.3)	χ² = 13.05	< 0.001*
Male sex, n (%)	12 (57.1)	58 (54.2)	χ² = 0.06	0.802
Five-minute Apgar score <7, n (%)	9 (42.9)	18 (16.8)	χ² = 7.37	0.007*
Resuscitation at birth, n (%)	11 (52.4)	26 (24.3)	χ² = 6.98	0.008*
Meconium‑stained fluid, n (%)	5 (23.8)	14 (13.1)	χ² = 1.63	0.202

Table 4 depicts the multivariate logistic regression analysis for independent predictors of early-onset neonatal septicemia. Prolonged membrane rupture was identified as the strongest independent predictor, with an adjusted odds ratio of 4.67 (p < 0.001). Intrapartum fever had an adjusted odds ratio of 3.89 (p = 0.003), whereas preterm delivery had an adjusted odds ratio of 3.21 (p = 0.005). Low birth weight and a five-minute Apgar score < 7 were also found to be significant independent predictors, with adjusted odds ratios of 2.98 and 2.42, respectively.

**Table 4:** Multivariate logistic regression analysis of independent predictors of early onset neonatal septicemia. Data were presented as adjusted odds ratios with 95% confidence intervals using multivariate logistic regression analysis. *p < 0.05 was considered statistically significant.

Variable (reference)	Adjusted odds ratio (aOR)	95% CI	p‑value
Preterm delivery (<37 weeks)	3.21	(1.42 - 7.26)	0.005*
Prolonged rupture of membranes (>18 h)	4.67	(2.05 - 10.64)	<0.001*
Intrapartum fever (≥38°C)	3.89	(1.56 - 9.68)	0.003*
Low birth weight (<2500 g)	2.98	(1.27 - 6.98)	0.012*
Five-minute Apgar score <7	2.42	(1.02 - 5.74)	0.045*

Table 5 illustrates the outcomes of neonates with and without early-onset neonatal septicemia. Mechanical ventilation was required in 11 neonates (52.4%) in the early-onset neonatal septicemia group compared to 18 neonates (16.8%) in the non-early-onset neonatal septicemia group (p < 0.001). Vasopressor support was needed in seven neonates (33.3%) with early-onset neonatal septicemia and eight neonates (7.5%) without early-onset neonatal septicemia (p = 0.001). In-hospital mortality was significantly higher among neonates with early-onset neonatal septicemia than among those without early-onset neonatal septicemia (p = 0.023). Shock and disseminated intravascular coagulation were also significantly more common in the early-onset neonatal septicemia group.

**Table 5:** Comparison of neonatal outcomes between neonates with and without early onset neonatal septicemia. Data were presented as median (interquartile range, IQR) or frequency and percentage. The Mann-Whitney U test was used for non-parametric continuous variables, and the chi-square test and Fisher’s exact test were used for categorical variables. *p < 0.05 was considered statistically significant; n = number of neonates; χ² = chi-square test.

Outcome	Early onset neonatal septicemia (n = 21), n (%)	Non-early-onset neonatal septicemia (n = 107), n (%)	Test statistic	p‑value
Length of neonatal intensive care unit stay (days), median (IQR)	18 (12 - 28)	9 (6 - 14)	Mann-Whitney U = 491	<0.001*
Need for mechanical ventilation, n (%)	11 (52.4)	18 (16.8)	χ² = 12.11	<0.001*
Need for vasopressors, n (%)	7 (33.3)	8 (7.5)	χ² = 10.61	0.001*
In‑hospital mortality, n (%)	4 (19.0)	5 (4.7)	χ² = 5.16	0.023*
Shock	6 (28.6)	8 (7.5)	χ² = 8.00	0.005*
Necrotizing enterocolitis	2 (9.5)	2 (1.9)	Fisher’s	0.108
Disseminated intravascular coagulation	3 (14.3)	1 (0.9)	Fisher’s	0.008*

## Discussion

Early-onset neonatal septicemia remains a major contributor to neonatal morbidity and mortality. This retrospective study evaluated the prevalence, maternal risk factors, neonatal characteristics, independent predictors, and clinical outcomes associated with early-onset neonatal septicemia in neonates admitted to a tertiary care hospital. The study demonstrated that 21 neonates (16.4%) developed early-onset neonatal septicemia, indicating a considerable disease burden in the study population. This prevalence is comparable to the findings reported by Simonsen et al. [1] and Shane et al. [13], who highlighted that neonatal sepsis continues to be a significant healthcare challenge in intensive care settings, especially among preterm and low-birth-weight neonates. The relatively high prevalence observed in the present study may be attributed to the inclusion of high-risk neonates managed in a tertiary referral center, where critically ill and premature neonates are commonly admitted.

The present study identified several maternal risk factors that are significantly associated with early-onset neonatal septicemia, including preterm delivery, prolonged membrane rupture, intrapartum fever, and clinical chorioamnionitis. Prolonged rupture of membranes was observed in 14 mothers (66.7%) in the early-onset neonatal septicemia group and emerged as the strongest independent predictor in multivariate analysis. Prolonged membrane rupture increases the duration of fetal exposure to ascending vaginal microorganisms, thereby facilitating intrauterine infection and neonatal bacterial colonization. Similar findings have been reported by Al-Lawama et al. [14] and Makhoul et al. [15], who demonstrated that prolonged membrane rupture significantly increased the risk of neonatal sepsis due to ascending maternal genital tract infections.

Intrapartum fever was another significant maternal predictor associated with early-onset neonatal septicemia in the present study. Maternal fever during labor often reflects an underlying intra-amniotic infection or systemic maternal inflammation, which can directly affect the fetus through the hematogenous spread of pathogens and inflammatory mediators. Clinical chorioamnionitis was also significantly associated with neonatal sepsis, supporting the established evidence that maternal intrauterine infections substantially increase neonatal infectious morbidity. These findings are consistent with those of a previous study by Tita and Andrews [16], who emphasized a strong relationship between maternal infectious conditions and adverse neonatal outcomes, including neonatal sepsis and respiratory complications.

Preterm delivery was significantly more common among neonates with early-onset neonatal septicemia. Premature neonates exhibit immature immune responses, reduced neutrophil storage pools, impaired skin and mucosal barriers, and lower transplacental transfer of maternal immunoglobulins, which make them highly susceptible to systemic infections. Similar observations have been documented by Stoll et al. [17], who reported a significantly increased susceptibility to sepsis among preterm neonates admitted to neonatal care units.

The neonatal characteristics evaluated in the present study also demonstrated significant associations with early-onset neonatal septicemia. Low birth weight was observed in 17 neonates (81.0%) with early-onset neonatal septicemia and was identified as an independent predictor in regression analysis. Low-birth-weight neonates often require prolonged hospitalization, invasive procedures, and respiratory support, all of which increase their vulnerability to infection. Additionally, low birth weight is frequently associated with premature birth and poor neonatal physiological reserves. Similar findings were reported by Camacho-Gonzalez et al. [18], who demonstrated that low birth weight is one of the strongest neonatal predictors of sepsis-related morbidity and mortality.

A five-minute Apgar score below seven and the need for resuscitation at birth were also significantly associated with early-onset neonatal septicemia [10,11,19]. Perinatal asphyxia and birth-related stress can impair neonatal immune defense mechanisms and increase tissue hypoxia, predisposing neonates to systemic infection. Neonates that require extensive resuscitation are also exposed to invasive interventions, which may increase the risk of infection. These findings align with those of studies conducted by Edmond and Zaidi [20], who reported an increased incidence of sepsis among neonates with birth asphyxia and poor Apgar scores.

The outcomes of neonates with early-onset neonatal septicemia were significantly poorer than those of neonates without sepsis. Neonatal sepsis can rapidly progress to septic shock, respiratory failure, disseminated intravascular coagulation, and multiorgan dysfunction owing to immature neonatal immune responses and delayed clinical recognition. The significantly longer duration of neonatal intensive care unit stay among neonates with sepsis observed in the present study further reflects the increased disease severity and healthcare burden associated with neonatal sepsis. The prolonged hospital stay observed among neonates with early-onset neonatal septicemia may be attributable to greater disease severity, prolonged antimicrobial therapy, need for respiratory and hemodynamic support, closer clinical monitoring, and management of sepsis-related complications. Similar findings have been reported by Fleischmann-Struzek et al. [21], who documented increased mortality, prolonged hospitalization, and higher complication rates among neonates with sepsis admitted to intensive care units.

Our findings have several important clinical implications. Early identification of maternal and neonatal risk factors, such as prolonged rupture of membranes, intrapartum fever, preterm delivery, low birth weight, and low Apgar scores, may facilitate prompt screening and timely initiation of empirical antimicrobial therapy in high-risk neonates. Strengthening antenatal care, improving intrapartum infection control measures, timely administration of intrapartum antibiotic prophylaxis, and close neonatal monitoring may reduce the burden of early-onset neonatal septicemia and improve neonatal survival outcomes in tertiary care settings.

The present study adds region-specific data on early-onset neonatal septicemia from a tertiary care neonatal intensive care unit over a five-year period and comprehensively evaluates maternal risk factors, neonatal predictors, and short-term clinical outcomes within a single analytical framework. Unlike studies limited to culture-proven sepsis, the present study additionally included probable sepsis cases identified through clinical features and sepsis screening parameters, thereby reflecting real-world neonatal intensive care practice where culture-negative sepsis remains common. The identification of independent predictors such as prolonged rupture of membranes, intrapartum fever, preterm delivery, and low birth weight may aid in early risk stratification and timely management of high-risk neonates, particularly in resource-limited tertiary care settings.

However, this study had some limitations. The retrospective design depended on the accuracy and completeness of available medical records, which may have introduced information bias. The study was conducted in a single tertiary care hospital with a small sample size, which limits the generalizability of the findings to other healthcare settings. Additionally, microbiological profiling and antimicrobial resistance patterns of the isolated organisms were not extensively evaluated. The clinical manifestations of early-onset neonatal septicemia are often nonspecific and variable, and retrospective assessment based on documented clinical records may not have captured the complete spectrum of presenting features in all neonates. Maternal Group B *Streptococcus* colonization status was unavailable for a substantial proportion of cases because routine antenatal screening was not consistently performed during the study period, which may have limited evaluation of its association with early-onset neonatal septicemia. The inclusion of both inborn and outborn neonates may also have introduced potential confounding because referred neonates could have differed in pre-referral clinical status, prior antibiotic exposure, and timing of treatment initiation, which were not uniformly available for retrospective evaluation. Future multi-center studies should be conducted on a larger sample size.

## Conclusions

Early-onset neonatal septicemia remains a significant cause of morbidity and mortality among neonates admitted to the neonatal intensive care unit. Maternal factors such as prolonged rupture of membranes, intrapartum fever, and preterm delivery, along with neonatal factors including low birth weight and Apgar scores, were significantly associated with early-onset neonatal septicemia. Neonates with sepsis demonstrate poorer clinical outcomes, including an increased need for intensive supportive care and a higher mortality rate. Early identification of high-risk neonates and timely intervention may improve neonatal outcomes and reduce disease burden.

## Appendices

Appendix 1: Structured data collection form

Section A. Record Identification

**Table 6:** Record identification.

Variable	Recorded value
Study ID	
Hospital Registration Number	
Date of Admission	
Date of Birth	
Inborn / Outborn	

Section B. Maternal Variables

**Table 7:** Maternal variables.

Variable	Recorded value
Maternal Age (years)	
Parity	
Gestational Age at Delivery (weeks)	
Mode of Delivery (Vaginal/Cesarean)	
Preterm Delivery (Yes/No)	
Prolonged Rupture of Membranes >18 hours (Yes/No)	
Intrapartum Fever ≥38°C (Yes/No)	
Clinical/Histopathological Chorioamnionitis (Yes/No)	
Maternal Urinary Tract Infection (Yes/No)	
Group B Streptococcus Colonization (Positive/Negative/Unknown)	
Intrapartum Antibiotic Prophylaxis (Yes/No)	
Maternal Diabetes Mellitus (Yes/No)	
Hypertensive Disorders of Pregnancy (Yes/No)	

Section C. Neonatal Variables

**Table 8:** Neonatal variables.

Variable	Recorded value
Sex	
Birth Weight (g)	
Low Birth Weight (<2500 g) (Yes/No)	
Very Low Birth Weight (<1500 g) (Yes/No)	
Extremely Low Birth Weight (<1000 g) (Yes/No)	
Apgar Score at 1 Minute	
Apgar Score at 5 Minutes	
Resuscitation at Birth (Yes/No)	
Perinatal Asphyxia (Yes/No)	
Meconium-Stained Amniotic Fluid (Yes/No)	

Section D. Early-Onset Neonatal Septicemia Assessment

**Table 9:** Early-onset neonatal septicemia assessment.

Variable	Recorded value
Clinical Signs Present Within 72 Hours (Respiratory distress/Poor feeding/Lethargy/Jaundice/Temperature instability)	
Blood Culture Performed (Yes/No)	
Blood Culture Result	
Organism Identified	
Total Leukocyte Count	
Absolute Neutrophil Count	
Immature-to-Total Neutrophil Ratio	
C-Reactive Protein (mg/L)	
Diagnosis: Proven Sepsis / Probable Sepsis / No Sepsis	

Section E. Clinical Outcomes

**Table 10:** Clinical outcomes.

Variable	Recorded value
NICU Stay Duration (days)	
Mechanical Ventilation Required (Yes/No)	
Vasopressor Support Required (Yes/No)	
Shock (Yes/No)	
Disseminated Intravascular Coagulation (Yes/No)	
Necrotizing Enterocolitis (Yes/No)	
In-Hospital Mortality (Yes/No)	
Final Outcome (Discharged/Expired/Transferred)	
